# Negro Traits

**Published:** 1902-07

**Authors:** 


### EDITORIAL NOTES AND COMMENTS.

The Business office of THE JOURNAL-RECORD is No. 64 Marietta Street.

The Editorial office is 318-319-320 The Prudential.

Address all Business communications to Dr. M. B. Hutchins, Mgr.

Make remittances payable to THE ATLANTA JOURNAL-RECORD OF MEDICINE.

On matters pertaining to the Editorial and Original Communications address Dr. Bernard Wolff, Atlanta.

Reprints of original articles will be furnished at cost price. Requests for the same should always be made *on the manuscript*.

We will present, post-paid, on request, to each contributor of an original article, twenty (20) marked copies of THE JOURNAL-RECORD containing such article.

### NEGRO TRAITS.

THE article which appeared in this journal some months ago on "Negro Traits," and contributed by Dr. E. G. Ferguson of Macon, has moved to speech some of our colleagues in the North and West. The editor of the *Detroit Medical Journal* was especially fervid in his criticism of the article. In this issue may be found Dr. Ferguson's rejoinder. Without reopening a question which seems destined never to be satisfactorily adjusted, it may be said that Dr. Ferguson's estimate of the negro character is in the main correct and coincides with the observation of all those who have been familiar with the ways of negroes by residence in that part of the country where they are most numerous. Dr. Ferguson's remarks we take to apply with especial force and accuracy to the Guinea negro, who is perhaps the type most frequently seen in the South.

It may be interesting in this connection to include the description of the Guinea negro by a French traveler and writer, Girard de Rialle. He describes him as being nearly always below medium height; the skeleton is powerful; the spinal column shows the three curves less than in the white man; the legs are bowed, making the lower extremities relatively longer than the upper; the foot is flat, the heel elongated; the shoulders are not very heavy, but the neck is short and powerful; the skull is ordinarily dolichocephalic, but among some individuals and tribes it is mesaticephalic and sub-brachiocephalic. The cranial capacity of the negro is sensibly less than in the white races. The occipital region is greatly developed; the frontal region on the contrary very slightly so. The superciliary ridges are but slightly prominent. The eyes are rather close together. The nose is flat, scarcely projecting above the level of the face. The teeth are white and sound. Prognathism is marked in the whole face, or only in the sub-nasal region, in which case the chin is flying. The pilous system is poorly developed. The hair of the scalp is short, black, woolly, and covers the whole head uniformly. The body is glabrous, except in the pubic and axillary regions. The eyes are black, with yellowish sclerotics; dark spots are found on the tongue, velum palati and conjunctiva; while on the contrary the hue is lightest on the palms of the hand and soles of the feet. The skin elsewhere tends toward an intense black, but may be reddish, yellowish, or even bluish. The negro is a grown-up child, swayed by the impulses of the moment, and the absolute slave to his passions. He is quick, inconsistent, happy and smiling, pleasure-loving, fond of dancing and such amusements. Vain to excess, he experiences the need of a master to speak with authority and make him feel the master's superiority. He is, nevertheless, familiar with every one, and has no real pride. He is hospitable and willing to share all he possesses with his friends. He is vindictive and exceedingly superstitious. He freely prostitutes his women from hospitality or for love of money. He is fetishistic and dominated by fear of demons. This account coincides with Dr. Ferguson's estimate of the negro.

Miscegenation has created a type to itself, the mulatto, who partakes of the characteristics of both bloods to the detriment of the white, and shows marked physical deterioration and lessened resistance to disease. However, such persons of color sometimes display mental qualities consistent with their white blood, and some have attained prominence in various directions. Dumas *père* is an instance in point. Booker Washington, the present leader of the negro in this country, is the royal bantling of a black mother and a white father. But these are "sports," exceptions, the great mass toeing the prescribed line of racial capability and trait. Occasionally a pure blood negro detaches himself from the crowd, but even in the enlarged perspective he remains the unaltered negro, and makes of himself a spectacle that excites more pity than admiration, as in the unhappy fate of the chauvinistic Toussaint L'Ouverture.

It is an impossible task for a man of American birth and ancestry to discuss the negro question dispassionately because this question was the occasion of the nation's greatest tragedy, the effects of which and the mental attitude caused by it still exist. Prejudice and prepossession even extend into ethnological considerations of the negro, so that attempts at scientific discussion are apt to be tinged by the writer's personal feeling in the matter. The subject had best be left alone and allowed to work itself out in accordance with that natural law which seems to forecast the doom of the negro. The implantation upon a primitive, crude and poorly resistant organism of the vices of the inured and sophisticated products of civilization, with the inevitable consequence of disease, is working toward the final extermination of the blacks. This is to the mind of the thinking men in the South the solution of the problem. It is the hand of fate, and argument on paper of theories for amalgamation and colonization are futile against it.

> "The moving finger writes, and having writ,
> Moves on, nor all your Piety and Wit
> Can lure it back to cancel half a line,
> Nor all your tears blot out a word of it."

